# Applying the science of measurement to biology: Why bother?

**DOI:** 10.1371/journal.pbio.3000338

**Published:** 2019-06-20

**Authors:** Carmen H. Coxon, Colin Longstaff, Chris Burns

**Affiliations:** National Institute of Biological Standards and Control, South Mimms, Potters Bar, Hertfordshire, United Kingdom

## Abstract

Both basic and translational research are continuously evolving, but the principles that underpin research integrity remain constant. These include rational, hypothesis-driven, and adequately planned and controlled science, which is carried out openly, honestly, and ethically. An important component of this should be minimising experimental irreproducibility. Biological systems, in particular, are inherently variable due to the nature of cells and tissues, as well as the complex molecules within them. As a result, it is important to understand and identify sources of variability and to strive to minimise their influence. In many instances, the application of metrology (the science of measurement) can play an important role in ensuring good quality research, even within biological systems that aren’t always amenable to many of the metrological concepts applied in other fields. Here, we introduce the basic concepts of metrology in relation to biological systems and promote the application of these principles to help avoid potentially costly mistakes in both basic and translational research. We also call on funders to encourage the uptake of metrological principles, as well as provide funding and support for later engagement with regulatory bodies.

## Abstract 2

Tackling reproducibility and improving research integrity are easier than you think; this Perspective article introduces the basic concepts of metrology in relation to biological systems and proposes that applying them could help avoid potentially costly mistakes in both basic and translational research.

## Metrology in biology

Irreproducibility in science is something that we will all have encountered at some point. It can be an issue when trying to reproduce experiments previously carried out by colleagues or fellow researchers or when repeating experiments using products or reagents from new or different suppliers. Although a source of great frustration, it is rare that academics have the luxury of spending months of dwindling grant time and an often-limited consumables budget to determine the underlying reason for these discrepancies. In fact, it is more likely that, driven by the need to publish and apply for more funding, work will continue regardless, and inappropriate conclusions may be drawn from the data. One reason for inaccurate or irreproducible reporting of data from biological experiments is the complexity of the biological systems being studied. For instance, problems can arise from a propensity to work in concentrations (e.g., mg/ml), often based on manufacturers’ recommendations or literature reports, without being mindful of the activity of the molecule or the complexity of the test system being employed. The activity of many biologicals may differ as a result of their production and manufacture; a milligram of enzyme or protein from manufacturer x will not necessarily have the same activity as a milligram of enzyme or protein from manufacturer y, and this can be important when this molecule is added to an experiment. What is required to ensure a reproducible experimental response is the addition of the same biological activity to the assay each time. Biological activity, usually but not always reported in units, can be derived through comparison with a specific reference preparation for that molecule to derive a relative activity rather than an absolute unit.

As an introduction to how the principles of metrology can apply to research, we will start with the analogy used by Philip Stark, who discussed the increasingly concerning issue of irreproducibility in scientific reporting. In his article [1], he makes the analogy of science experiments being like baking bread, highlighting the need for enough detail in the ‘recipe’ to allow others to make a ‘similar loaf’. One essential part of the recipe is the method—variations in timing, processing, and temperature can all influence the end product. The other key element here is your starting material (i.e., the precise description of the ingredients and their quantities). If these can be described accurately, then a reproducible result (delicious bread) is more likely. The analogy to scientific experimentation is clear, but in biological systems, it may not necessarily be easy, or indeed possible, to precisely define the method or measure the precise quantity of the ‘ingredients’.

Metrology, or the science of measurement, harmonises almost every facet of our lives to ensure we can communicate, trade, function, and work as a global community. This discipline is well established in the physical sciences, but the complexity of biological systems makes the application of many of the core principles of metrology much more challenging [2–4]. For instance, the metrological concept of measuring the true level of a given measurand (a quantity intended to be measured) is a fundamental tenet of measurement science, but it only works when you know exactly what it is you are measuring—i.e., the measurand can be clearly and unambiguously defined, such as the levels of oxygen in the atmosphere. Many biological samples are complex and of unknown composition such that they cannot be described in clear physical and chemical terms. This often means that measurements instead rely on an observable and measurable change in biological activity—for example, a measurable response from cultured cells proportional to the activity of a molecule, or collection of molecules, within a complex mixture. In many cases, it is not possible to precisely define the measurand and, by extension, trace these measurements in absolute terms to the International System of Units (SI)—for example, grams or kilograms. Tests to measure biological activity are comparative rather than absolute, and biological reference materials are critical in defining the relative magnitude of the biological response.

The use of biological reference materials and the application of metrology is relatively well established in the field of biological medicine manufacture. Well-characterised reference materials are used to generate comparability data in bridging studies to ensure that the final drug product is comparable to versions tested previously in the clinical programme. In this instance, the inclusion of suitable reference materials to assess the effect of manufacturing changes (e.g., scaling up, different cell lines, more rigorous purification methods, etc.) can obviate the requirement for additional clinical studies, reducing delays to licensure and the associated costs; such savings can have a huge impact on spin-out companies and small and medium enterprises. Similarly, reference materials can help to ensure that drug potency does not alter over subsequent manufacturing rounds or scaling up or that the same drug marketed by different manufacturers is of comparable potency [5]. However, these principles can be extended to basic research. Consider, as an example, mammalian cell culture, a model system commonly used throughout research laboratories. In many instances, these models are used as surrogates to investigate the whole-animal response to external stimuli. This may be to elucidate a signalling pathway in the cells or the mechanism of action of the given molecule. Many cell culture models employ cytokines, hormones, and growth factors as growth-promoting agents, many of which are produced using recombinant technology. The activity of these biological reagents is known to be dependent on their route of manufacture and can differ even between batches of the same product from a single manufacturer. For instance, differences in the posttranslational modification of biological molecules can have profound effects on their activity both in vitro and in vivo; examples include sialylation [6], oxidation [7], sulfation [8], and disulfide bond reduction/oxidation [9–12]. As a result, the addition of biological reagents to cell culture based on mass or concentration (i.e., mg/ml) may lead to inconsistent or irreproducible effects on the cells, which would then be an unknown and uncontrolled experimental variable. By carrying out a simple comparison with a reference material of known biological activity that is fit for purpose in the context of the assay, one can be more confident that the amount of biologically active material in each experiment is always the same, regardless of the source of the material.

Comparison with a reference material is an important aspect of metrology and is a practise that can be applied to biological systems. This type of comparison is an example of traceability and can also be exemplified in practise with the ruler. Most people have a 30-cm ruler, but how do you know it is truly 30 cm? The manufacturer of the ruler will have an ‘in-house’ calibrator or ‘standard’ for their production facility that can be traced all the way to the definitive international standard for the metre (defined as the length of the path travelled by light in vacuum in 1/299,792,458 of a second [https://www.bipm.org/en/CGPM/db/17/1/]). This traceability means that you can be confident your 30-cm ruler is the same length as every other ruler around the world. In the case of the activity of a biological material, traceability is realised by comparing it with a reference standard for which the biological activity is calibrated in arbitrary units (U). The U for a biological reference standard is unique to that material and, unlike the units of the SI, has no physical existence beyond the reference standard that defines it.

At the National Institute for Biological Standards and Control (NIBSC), we have been developing and globally distributing biological reference materials on behalf of WHO for decades. These materials promote harmonisation of research results, the manufacture of safe and effective medicines, and the implementation and harmonisation of clinical diagnostics across a broad range of biological disciplines. These reference materials have an assigned biological activity, usually defined in international units (IU) [13], derived by consensus following a collaborative study. Importantly, where technological advances permit, we use physicochemical methods to assign SI units to our reference materials (e.g., grams or moles) rather than IU. For example, reference materials for vitamins and antibiotics were originally assigned values in IU based on their activity in bioassays. However, as they became fully chemically characterised, gravimetric weight could be used. This is also the case for several peptide hormones, and molar concentrations have been estimated by active site titration for several haemostasis enzyme standards [14]. In many areas, such as vaccine development [15], clinical diagnostics, and the production of classical biological medicines (e.g., recombinant proteins or products derived from human blood), scientists are aware of the applicability of biological standards available from NIBSC and other standards-setting organisations. Given the central role these materials have in assuring the quality of medicines and ensuring that clinical trial data are robust and reproducible, it can be reasonably inferred that their limited use in basic and preclinical research is a contributing factor to the alarming cost of irreproducibility, which is estimated to be around US$28 billion per year in the United States alone [16]. Furthermore, in novel areas of drug discovery, such as cell [17, 18] and gene therapies [19], and the next generation of biotechnology products including antibody-based therapeutics and modified biologicals (e.g., extended half-life products), we are increasingly aware that there is a lack of recognition of the importance of reference materials, particularly among the research community. Considering the current reproducibility crisis, which is of concern to both the scientific and political communities [20], the implementation of reference materials to ensure that research and the development of medicines are robust and efficient processes is something we urgently need to address. Where reference materials are available, they should be incorporated into routine working practises. It is possible to establish an in-house reference material for routine use that is standardised to an international standard or to a reference material that is traceable to the international standard, and this is a common practise among manufacturers of biological therapeutics. If a standard does not exist, it is still possible to establish in-house reagents that can be used to ensure consistency between batches of material over the duration of a project. Help and guidance on these approaches can be sought from the standards organisations below.

## A little history

Using a biological reference material to quantitate biological activity is not a new concept. It may surprise many that standardisation of biological activity can be traced back to the 1890s, not long after the Treaty of the Metre was signed in 1875. Emil von Behring, working at the Robert Koch Institute in Berlin, discovered that serum extracted from horses inoculated with the diphtheria bacterium was effective in treating the infection in human patients. However, there was significant variability in the potency of these serum batches, which was addressed when Paul Ehrlich, working with Behring, established that the only way to accurately determine the potency of each batch was to express it in relation to a comparator serum preparation, or ‘standard’ [21]. This led to the establishment of the first IU for a biological substance, and today, WHO maintain a central role in biological standardisation through their Expert Committee on Biological Standardization, formed in 1947.

Biological standardisation is clearly important in the potency determination and clinical adoption of complex, difficult-to-characterise biological substances, but widespread acknowledgement of its utility is perhaps lacking. J. H. Humphrey, then president of the International Union of Immunological Societies, wrote letters to both the *Lancet* and the *BMJ* in 1976 that began, ‘Sir, until the value and importance of using International Standards has become generally accepted, it may seem necessary from time to time to remind the scientific community of their purpose and even, perhaps, of their existence’ [22]. It appears that this statement, written over 40 years ago, is still relevant today. He goes on to state that ‘The value of a unit is arbitrary but is chosen to be convenient for the purpose, and … provides, therefore, the one invariable quantity against which unknown materials can be evaluated using different tests in different laboratories.’

## Changing scientific working practises

Although some biological scientists are mindful of the importance of reference materials and traceability, we believe it is now timely to advocate and promote their wider use in routine research, when appropriate. For what would represent a relatively small change in research culture, the adoption of these principles may in fact be a significant step forward to address irreproducibility in research. Further to this—and, importantly, from an animal welfare perspective—reference materials can also play an essential role in the replacement of in vivo models with ex vivo or in vitro alternatives when appropriate and when there is a critical requirement to demonstrate comparability or bridging between assay types.

As stated in a recent commentary in *Nature Methods* [23], ‘the first step is communication between biologists and the measurement scientists’, a sentiment that has been echoed elsewhere [2, 3]. Many organisations are committed to engaging with the scientific community to provide biological reference materials that are fit for purpose and permit the comparability of data through space and time, as well as identify sources of experimental variability. Many biological reference materials and biological assays are available; however, where these do not exist, scientists should be encouraged to engage with these organisations to discuss their requirements (a number of these are listed in Table 1). We at NIBSC welcome discussion and collaboration on areas pertaining to improving public health and work in collaboration with academia and industry to provide materials to meet this challenge. Other standardisation bodies focus on more specific areas; for example, the Standards Coordinating Body (SCB) for Gene, Cell, and Regenerative Medicines and Cell-Based Drug Discovery, who support the identification of materials needed by the regenerative medicines community, facilitate their development, and promote their use through communication and education.

**Table 1:** Organisations developing and distributing standards and reference materials.

Organisation	Role/Mission	Areas
NIBSC (government)	Assures the quality of biological medicines through the provision of biological reference materials, by testing products (control), and by carrying out research. NIBSC also provide expert advice in response to emergencies as well as guidance to manufacturers, regulatory authorities, United Kingdom government and European bodies, the UN, and WHO.	Advanced therapies, vaccines, blood products, haemostasis, toxins, stem cells, gene therapy, next-generation sequencing, posttranslational modifications, biosimilars, cytokines and growth factors, microbiome.
NIST (government)	Nonregulatory government agency within the US Department of Commerce. Founded in 1901, it is the national measurement (metrology) institute for the US. NIST provides guidance to federal agencies on the participation in, and use of, voluntary written and physical standards.	Measures and weights, >1,200 standard reference materials for instrument calibration and method development, laboratory accreditation, documentary standards, standard reference data (e.g., biometrics, mass spectrometry), reference materials for nontargeted metabolomics, lipidomics, and proteomics.
EDQM	Development, implementation, and monitoring of quality standards (written and physical) for manufacture and control of safe medicines and cosmetics, coordination of European OMCLs, combating counterfeit drugs, guidance in safe use of medicines and cosmetics.	Blood transfusion; organ, cell, and tissue transplantation; biotherapeutics; consumer health issues; cosmetics and food contact materials; monographs and reference standards of the European Pharmacopoeia.
CEN	Provide voluntary European standards and related products and services for the benefit of businesses, consumers, and other standard users in Europe and drive agreement on common specifications and/or procedures that respond to the needs of business and meet consumer expectations.	Advanced manufacturing and processing, advanced materials, biotechnology, nanotechnologies, and nanomaterials (including healthcare and consumer products).
SCB	SCB aims to connect producers of written and physical standards with users of these materials and therefore promote their uptake and implementation. Mission: ‘Coordinate the accelerated advancement and improved awareness of the standards and best practices that address the rapidly evolving needs of the global regenerative medicine advanced therapy community’.	Cell-based drug discovery, cell therapy, gene therapy, tissue engineering, rapid microbial testing for commercial cell or gene therapy products, ancillary materials for cellular therapies, cell collection standards.
ARM	International organisation focused on regenerative medicine and advanced therapies. Works with members and policymakers to foster investment, research and development, and successful commercialisation of safe, effective, and transformational therapies. Enable market access and advocate clear regulatory pathways and procedures.	Cell therapy, gene therapy, and gene-modified cell therapy, tissue engineering and biomaterials, Europe/US market access and European/US regulatory affairs, patient engagement, European advanced therapies, communication and education.
NPL	The UK’s NMI, developing and maintaining the national primary measurement standards (physical). It is a public corporation owned by the Department of BEIS.	Drug discovery, manufacture and formulation, medical devices, tissue imaging, data analysis, biophysics, radiotherapy, environmental monitoring.
LGC	Provide a comprehensive range of reference materials, proficiency testing schemes, genomics reagents and instrumentation, research and measurement services. Also, manages and operates laboratories on behalf of the UK government to provide independent resolution of technical appeals in the UK food and feed enforcement system and give advice to government, the public sector, and the wider analytical community on measurement science particularly in relation to legislation and regulation.	Bioanalytical services pertaining to small molecules and biotherapeutics, CMC advice for drug manufacture, oligonucleotide therapeutics.
Pharmacopoeia (various, UK, US, China, Europe, etc.)	Publish written standards (monographs) for pharmaceutical substances and medicinal products; supported by physical reference materials. Can include veterinary substances and products. Provides a legal and scientific basis for quality control during the development, production, and marketing processes. Covers qualitative and quantitative composition, tests to be carried out on medicines, raw materials used in production of medicines, and on the intermediates of synthesis.	Written standards and physical reference materials for dosage forms, active pharmaceutical ingredients, formulated preparations and excipients, herbal drugs and herbal medicinal products, blood-related products, immunological products, radiopharmaceutical preparations, infrared reference spectra.

Abbreviations: ARM, Alliance for Regenerative Medicine; BEIS, Business, Energy, and Industrial Strategy; CEN, European Committee for Standardisation; CMC, Chemistry, Manufacture, and Control; EDQM, European Directorate for the Quality of Medicines and Healthcare; LGC, Laboratory of Government Chemists; NIBSC, National Institute for Biological Standards and Control; NIST, National Institute of Standards and Technology; NMI, National Metrology Institute; NPL, National Physics Laboratory; OMCL, Official Medicines Control Laboratory; SCB, Standards Coordinating Body

## Recommendations

Biological reference materials are likely to move away from their traditional application of potency measurements in animal models or cell-based assays to more basic research applications. Examples include standardisation of next-generation sequencing applications; flow cytometry technology; and, as discussed previously, reagents used in day-to-day research. We encourage the research community and, in particular, funding bodies to be mindful of the inclusion of reference materials in routine work and to consider how they can use these reagents before embarking on hypothesis testing [3]. NIBSC is one of several standards-setting agencies within both the WHO network of collaborating centres and the network of national metrology organisations, which form a framework for measurement science. These organisations, and others listed in Table 1, welcome any dialog that will help identify where we can use our expertise to improve scientific research and drug discovery and, ultimately, improve public health and quality of life. Let us hope that the next time J. H. Humphrey’s quote is mentioned, it is to celebrate how far we have come rather than to emphasise how little has changed.

## Abbreviations

ARM: Alliance for Regenerative Medicine
BEIS: Business, Energy, and Industrial Strategy
CEN: European Committee for Standardisation
CMC: Chemistry, Manufacture, and Control
EDQM: European Directorate for the Quality of Medicines and Healthcare
IU: international unit
LGC: Laboratory of Government Chemists
NIBSC: National Institute for Biological Standards and Control
NIST: National Institute of Standards and Technology
NMI: National Metrology Institute
NPL: National Physics Laboratory
OMCL: Official Medicines Control Laboratory
SCB: Standards Coordinating Body
SI: International System of Units
U: arbitrary unit
